# Mechanisms of Chemotherapy-Induced Peripheral Neuropathy

**DOI:** 10.3390/ijms20061451

**Published:** 2019-03-22

**Authors:** Renata Zajączkowska, Magdalena Kocot-Kępska, Wojciech Leppert, Anna Wrzosek, Joanna Mika, Jerzy Wordliczek

**Affiliations:** 1 Department of Interdisciplinary Intensive Care, Jagiellonian University, Medical College, 31-501 Krakow, Poland; surmiatko@interia.pl (A.W.); j.wordliczek@uj.edu.pl (J.W.); 2 Department of Pain Research and Treatment, Jagiellonian University, Medical College, 31-501 Krakow, Poland; makoco@wp.pl; 3 Chair and Department of Palliative Medicine Poznan University of Medical Sciences, 61-245 Poznan, Poland; wojciechleppert@wp.pl; 4 Institute of Pharmacology, Polish Academy of Sciences, Department of Pain Pharmacology, 31-343 Krakow, Poland; joasia272@onet.eu

**Keywords:** chemotherapy-induced neuropathy, cancer pain, drug neurotoxicity, pathophysiological mechanisms, anticancer drugs

## Abstract

Chemotherapy-induced peripheral neuropathy (CIPN) is one of the most frequent side effects caused by antineoplastic agents, with a prevalence from 19% to over 85%. Clinically, CIPN is a mostly sensory neuropathy that may be accompanied by motor and autonomic changes of varying intensity and duration. Due to its high prevalence among cancer patients, CIPN constitutes a major problem for both cancer patients and survivors as well as for their health care providers, especially because, at the moment, there is no single effective method of preventing CIPN; moreover, the possibilities of treating this syndrome are very limited. There are six main substance groups that cause damage to peripheral sensory, motor and autonomic neurons, which result in the development of CIPN: platinum-based antineoplastic agents, vinca alkaloids, epothilones (ixabepilone), taxanes, proteasome inhibitors (bortezomib) and immunomodulatory drugs (thalidomide). Among them, the most neurotoxic are platinum-based agents, taxanes, ixabepilone and thalidomide; other less neurotoxic but also commonly used drugs are bortezomib and vinca alkaloids. This paper reviews the clinical picture of CIPN and the neurotoxicity mechanisms of the most common antineoplastic agents. A better understanding of the risk factors and underlying mechanisms of CIPN is needed to develop effective preventive and therapeutic strategies.

## 1. Introduction

Cancer is currently a leading cause of mortality worldwide [1]. However, thanks to advances in medicine and modern technology, the availability of sensitive tests and diagnostic methods to detect cancer at an early stage and the use of increasingly effective treatments, including chemotherapeutic agents, the number of cancer survivors is rising: It is expected to increase by 35%, from 13.7 million in 2012 to 18 million, by 2022 [2]. Although these survivors may have beaten cancer, many of them have poor outcomes due to a number of syndromes that reduce the quality of life as a consequence of cancer treatment, including pain, which they often experience for a long time after completing their cancer treatment [3].

Drugs used in cancer chemotherapy constitute an extremely effective tool in arresting the progression of cancer since they have numerous targets and mechanisms of action aimed at eliminating rapidly dividing cancer cells. Unfortunately, these drugs also affect normal cells and structures of the body, causing various deleterious and sometimes even devastating side effects (e.g., anaemia, diarrhea, nausea, vomiting, infections, neurological changes, fatigue, hair loss, infertility, pain and peripheral neuropathy) [4], which may necessitate the tapering of chemotherapy regimens or even their cessation, thereby limiting the efficacy of cancer treatment.

Chemotherapeutic agents can damage nervous system structures and, depending on the individual compound, can cause a variety of neuropathies: large and small fibre, sensory and/or motor, demyelinating and axonal, cranial and autonomic [5]. The effects of chemotherapy on the nervous system vary among the different classes of drugs, depending on the specific physical and chemical properties of the drug used and its single or cumulative doses [6]. One of the most common neuropathies caused by antineoplastic agents is a condition known as chemotherapy-induced peripheral neuropathy (CIPN) [7]. The prevalence of CIPN is agent-dependent, with reported rates varying from 19% to more than 85% [8] and is the highest in the case of platinum-based drugs (70–100%), taxanes (11–87%), thalidomide and its analogues (20–60%), and ixabepilone (60–65%) [6]. Toxicity may occur either with a high single dose or after cumulative exposure. Observed symptoms vary in intensity and duration and range from acute, transient thermal sensations to permanent changes in peripheral nerves accompanied by chronic pain and irreversible nerve damage [9]. Recent studies put the prevalence of CIPN at approximately 68.1% when measured in the first month after chemotherapy, 60.0% at 3 months, and 30.0% at and after 6 months [9].

CIPN is a predominantly sensory neuropathy that may be accompanied by motor and autonomic changes [9]. Except for paclitaxel and oxaliplatin, which cause acute neuropathy during or immediately after infusion [10], CIPN symptoms usually emerge late, that is, weeks or months after the completion of chemotherapy, with their severity being usually proportional to the cumulative dose of the drug [11]. Some patients experience paradoxical worsening and/or intensification of symptoms after the cessation of treatment [12], as well as a phenomenon known as “coasting”, where either mild neuropathy worsens or new CIPN develops. This situation poses a challenge for oncologists since, during the chemotherapy course, no signs or indications warrant a reduction in the dosage to mitigate CIPN symptoms [13]. Pain and sensory abnormalities may persist for months or even years after the cessation of chemotherapy. Therefore, patients may be cancer-free but may suffer from debilitating neuropathy induced by cancer treatment [14].

Clinically, CIPN manifests itself as deficits in sensory, motor and/or autonomic functions of a varying intensity [15]. Sensory symptoms usually develop first, involve the feet and hands and commonly present as a typical “glove and stocking” neuropathy with the most distal parts of the limbs exhibiting the greatest deficits. The symptoms comprise numbness, tingling, altered touch sensation, impaired vibration, paresthesias and dysesthesias induced by touch and warm or cool temperatures. Moreover, painful sensations, including spontaneous burning, shooting or electric shock-like pain as well as mechanical or thermal allodynia or hyperalgesia frequently occur [16]. In severe cases, these symptoms can progress to a loss of sensory perception. Motor symptoms occur less frequently than sensory symptoms and, as a rule, assume the form of distal weakness, gait and balance disturbances, and impaired movements. These symptoms have a marked and often underappreciated impact on quality of life and safety, e.g., cancer patients who develop CIPN are three times more likely to fall [17]. In severe cases, CIPN can lead to paresis, complete patient immobilization and severe disability [18]. Sensory disorders occur more frequently than autonomic symptoms, which usually involve orthostatic hypotension, constipation and altered sexual or urinary function [18].

In comparison to other peripheral neuropathies, for instance painful diabetic polyneuropathy, patients with CIPN may present more fulminant symptoms, affecting at the same time the feet and hands, with predominant pain, and symptoms have a faster progression as well. According to findings coming from electrodiagnostic studies, CIPN may be characterized as an axonal sensorimotor neuropathy, while painful diabetic neuropathy may be classified as a mixed neuropathy [19].

CIPN is perceived by many clinicians as a side effect of life-saving or at least life-prolonging therapy, which, due to its positive impact on a patient’s future fate, is deemed acceptable. However, many patients judge it primarily from the perspective of extremely unpleasant complaints, which cause suffering and hence significantly reduce the quality of life in the intervening years [20]. Given the potential chronicity of chemotherapy-induced biochemical and cellular changes, oncologists involved in chemotherapy should be aware of the magnitude and seriousness of the problem, should know the factors that increase the risk of CIPN and should be aware of the fact that cancer survivors may require a lifetime of medical monitoring and treatment of drug-induced health problems and comorbidities [21]. It is very important, especially in the case of platinum-based anticancer agents and taxanes; with these drugs, CIPN may last several years after the completion of chemotherapy [22].

A number of predisposing risk factors of CIPN have been identified, including patient age (higher risk in older patients); the co-occurrence of neuropathy before the start of chemotherapy (e.g., diabetic neuropathy); a history of smoking; impaired renal function with reduced creatinine clearance; exposure to other neurotoxic chemotherapeutic agents; paraneoplastic antibodies; and independent, direct cancer-associated neuropathy. Genome-wide association studies (GWAS) identified some single nucleotide polymorphisms (SNPs) associated with a higher risk of CIPN. The reported polymorphisms are associated with a range of proteins, including voltage-gated sodium channels, Schwann cell function–related proteins, receptors for cell surface collagen, receptors involved in neuronal apoptosis, neuronal crest cell development and an enzyme involved in pyruvate metabolism [9]. The cumulative dose of chemotherapeutic agents is another well-recognized major risk factor of CIPN [5,9,14].

Chemotherapeutics exerting neurotoxic effects on the peripheral nervous system are used as standard, routine medications against the most common types of cancer. Six main agent groups cause damage to the peripheral sensory, motor and autonomic neurons, resulting in CIPN development: platinum-based antineoplastics (particularly oxaliplatin and cisplatin), vinca alkaloids (particularly vincristine and vinblastine), epothilones (ixabepilone), taxanes (paclitaxel, docetaxel), proteasome inhibitors (bortezomib) and immunomodulatory drugs (thalidomide) [12]. Among them, the most neurotoxic classes of anticancer drugs are platinum-based drugs, taxanes, ixabepilone and thalidomide and its analogues; other, less neurotoxic but also commonly used drugs are bortezomib and vinca alkaloids.

The pathomechanism by which chemotherapeutics damage the nervous system structures and cause CIPN is multifactorial and involves microtubule disruption, oxidative stress and mitochondrial damage, altered ion channel activity, myelin sheath damage, DNA damage, immunological processes and neuroinflammation [23]. In the subsequent part of this paper, we review the clinical picture of CIPN and the exact neurotoxicity mechanisms associated with individual drugs most commonly used in cancer chemotherapy, namely, platinum-based antineoplastics, immunomodulatory drugs (thalidomide), taxanes, epothilones (ixabepilone), vinca alkaloids and proteasome inhibitors (bortezomib).

## 2. Platinum-Based Antineoplastics (Oxaliplatin, Cisplatin and Carboplatin)

Platinum-based chemotherapeutic agents (oxaliplatin, cisplatin and carboplatin) are widely used in the treatment of several types of solid tumors. Oxaliplatin is indicated for the treatment of digestive tract tumors (advanced colorectal, esophageal, stomach, liver and pancreatic cancers), while cisplatin and carboplatin are indicated for the treatment of other types of tumors (small-cell lung cancer, testicular, ovarian, brain, uterine and bladder cancers). Acute and chronic neurotoxicity following platinum-based chemotherapy is a major limitation, contributing to prolonged infusion times, dose reductions, treatment delays or even the cessation of treatment [24]. In addition to peripheral neuropathy, cisplatin may also induce ototoxicity, myelotoxicity and nephrotoxicity. Cisplatin-induced peripheral neuropathy (CisIPN) occurs in a time- and dose-dependent manner. The onset of neuropathy may be variable, with some patients reporting onset of symptoms after the first dose and some reporting onset after 12 cycles of therapy [25]. CisIPN develops after cumulative doses above 350 mg/m^2^, and at the cumulative dose of 500–600mg/m^2^, CisIPN occurs in 92% of patients [26]. Epidemiological data show that the incidence of neuropathic symptoms for cisplatin ranges from 49% to 100%, while chronic CisIPN has been observed in 5–20% of patients at 12 months after therapy [15,27]. The severity of CisIPN and the likelihood of chronicity increases with higher cumulative doses and longer exposure times to cisplatin [28]. The development of CisIPN seems to be independent of pretreatment, age, sex, tumor type and cotreatment with other chemotherapeutics [15,29]. Carboplatin seems to be less toxic, with neuropathy observed in 13–42% of patients [30]. Therapy with oxaliplatin may induce side effects such as myelotoxicity and enteric and peripheral neuropathy (OIPN, oxaliplatin-induced peripheral neuropathy). Acute, transient OIPN develops in almost 65–98% of patients within hours of oxaliplatin infusion at a dose ranging from 85 to 130 mg/m^2^ and may last up to 5–7 days. In patients receiving 12 cycles of chemotherapy, symptoms may persist up to 21 days or longer [31,32]. The symptoms of acute OIPN include cold-related paresthesias of the hands and feet, pharyngolaryngeal dysesthesias, jaw spasms, fasciculations and muscle cramps [33]. Cold-induced neuropathy after oxaliplatin is a unique feature of OIPN, and this is the most important difference in the clinical presentation between oxaliplatin and cisplatin-induced neuropathy [34]. Attal et al. have shown that the duration of cold- (and touch-) evoked pain reported during the first three cycles was associated with the extent of the chronic form of OIPN experienced one year later [35]. The chronic form, described as a pure sensory, axonal neuropathy, with a classical stocking-and-glove distribution, is observed in 50–70% of patients, but the incidence depends on the time point after oxaliplatin treatment and the intensity of symptoms assessed [33,36,37,38]. According to data presented in a systematic review by Beijers et al. [39], OIPN may be present in 26–46% of patients at the 12-month follow-up, in 24% of patients at the 15–18-month follow-up or even in 84% of patients at the 24-month follow-up, which has been shown in the study of Briani et al. [40].

The most important risk factors of acute and chronic OIPN include the cumulative oxaliplatin dose, the 2 h time of infusion, low body weight, younger age, a body surface area > 2,0, gene variations (GSTP1, glutathione-S-transferase genes P1; GSTM1, glutathione-S-transferase genes M1; and voltage-gated sodium channel genes SCN4A, SCN9A and SCN10A) and peripheral neuropathy symptoms prior to chemotherapy [41,42,43,44]. High-grade chronic OIPN occurs in approximately 10% of patients receiving cumulated doses ranging from 510 to 765 mg/m^2^, while at doses higher than 1000 mg/m^2^, this condition may be present in almost 50% of patients [45]. It is known that the acute form of OIPN is also a risk factor for chronic OIPN; a higher intensity of the acute form results in a higher incidence of chronic neuropathy [10].

The antineoplastic mechanisms of platinum-based chemotherapeutic action include the following [46,47,48,49,50]:

- The binding to nuclear DNA (deoxyribonucleic acid) by cancer cells and the formation of DNA-platinum adducts, resulting in the inhibition of DNA replication and RNA (ribonucleic acid) transcription, followed by the arrest of cancer cell division, with the DNA adducts activating apoptotic pathways that induce cell death and tumor degradation;
- The alteration of mitochondrial function followed by the disruption of the respiratory chain function and an increased production of reactive oxygen species (ROS);
- The inhibition of mitochondrial DNA replication and transcription, leading to an altered mitochondrial function and the activation of apoptosis;
- The activation of the immune system (macrophages, T-cells and monocytes) followed by the release of pro-inflammatory cytokines and the activation of apoptosis;
- The influence on calcium signaling pathways and the function of protein kinase families (MAPK, mitogen activated protein kinases; JNK, c-Jun N-terminal kinase; PKC, protein kinase C; AKT, serine-threonine kinases), leading to tumor cell apoptosis.

**Figure 1:**
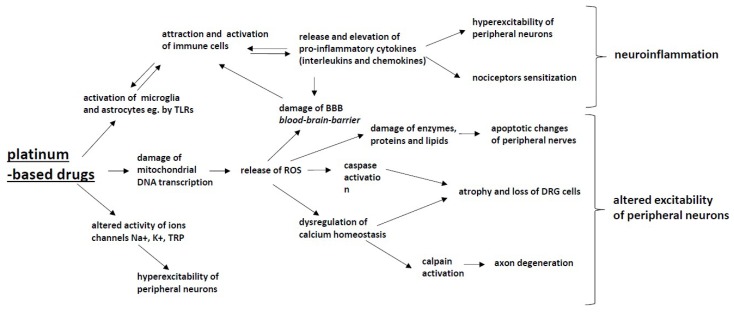
The mechanisms of chemotherapy-induced peripheral neuropathy (CIPN) induced by platinum-based drugs: Platinum-based drugs induce the activation of glia cells, which leads to the activation of the attraction and activation of immune cells and to the release and elevation of pro-inflammatory cytokines (interleukins and chemokines), which results in nociceptor sensitization and hyperexcitability of peripheral neurons, and (together with ROS) damage the blood–brain barrier. These processes lead to the development of neuroinflammation. Mitochondrial damage caused by platinum-based drugs leads to an increased production of reactive oxygen species (ROS), which leads to enzyme, protein and lipid damage within neurons as well as the dysregulation of calcium homeostasis, which induces apoptotic changes in peripheral nerves and in DRG cells. Platinum-based drugs also alter the activity Na+, K+ and TRP ion channels, resulting in the hyperexcitability of peripheral neurons. All of the above-described processes have the potential to alter the excitability of peripheral neurons.

The exact mechanism of peripheral neuropathy induced by platinum-based chemotherapeutics is not yet fully understood; however, it seems that their antitumor mechanisms are responsible for the neurotoxic effect, since chemotherapeutics induce numerous changes either in the structure or functioning of neuronal and glial cells [51]. Chemotherapeutic agents induce several alterations in intracellular organelles (particularly mitochondria), membrane receptors and ion channels, followed by alterations of the intracellular homeostasis, signaling and neurotransmission, all of which may result in neuroinflammation, DNA damage and axonal degeneration (Figure 1).

The unique feature of oxaliplatin is rapid nonenzymatic transformation to reactive platinum complexes and leaving-group oxalate. Oxalate has been proposed to contribute to acute cold-induced neuropathy, which is most characteristic of oxaliplatin treatment. The study performed in laboratory animals by Pereira et al. showed that either oxaliplatin or its oxalate-free cytotoxic analogue induced peripheral sensory neuropathy, although oxalate led to a partial and later decrease in mechanical threshold in comparison with oxalate-free analogue [52].

Platinum agent-induced peripheral neuropathy is initiated by the accumulation of platinum adducts in dorsal root ganglion (DRG) and trigeminal ganglion (TG) neurons. This process is probably the major mechanism responsible for the initiation of neurotoxicity induced by this class of chemotherapeutics [53,54,55]. The results of Fujita et al. indicate that oxaliplatin transporters Octn1 and Mate1 are involved in platinum accumulation in DRG neurons, followed by OIPN. The accumulation of platinum has been observed in cells overexpressing Octn1 and Mate1, which are correlated proportionally with the severity of neuropathic behavior in laboratory animals [56].

The exact mechanism of platinum-based agent neurotoxicity in humans is still discussed; however, experimental and preclinical studies have provided insight into the processes most likely involved in neuropathy pathogenesis. Some of these mechanisms are discussed in more detail below.

### 2.1. Mitochondrial Dysfunction and Oxidative Stress

Mitochondrial dysfunction and oxidative stress have been highlighted as key players in the pathophysiology of platinum-induced neuropathy. After entering neuronal and nonneuronal cells, oxaliplatin and cisplatin bind to mitochondrial DNA (mDNA) and form mDNA adducts. These pathological products cannot be repaired because there is no DNA repair system in mitochondria. Platinum-mDNA adducts impair the physiological replication and transcription of mDNA, which may lead to the synthesis of abnormal proteins, resulting in the impairment of the respiratory chain in mitochondria [48,53,57,58,59]. The impairment of the mitochondrial physiological function leads, in turn, to decreased cellular metabolism, to an increased production of ROS (reactive oxygen species) and to oxidative stress [60,61,62,63]. Imai et al. showed that cisplatin and oxaliplatin may induce mitochondrial dysfunction in cultured Schwann cells [64]. Oxaliplatin was shown to significantly increase superoxide anion production and to induce lipid peroxidation, protein and DNA oxidation in both sciatic nerves and the spinal cord in an in vitro study [65]. Further inhibition of cellular metabolism and pathological, high intracellular levels of ROS may, in turn, lead to damage to enzymes, proteins and lipids, resulting in structural changes of peripheral nerves [66]. Oxidative stress followed by apoptotic changes has been observed in the sciatic nerves of cisplatin-treated mice [67].

ROS can also activate the apoptotic pathway in neuronal cells through mitochondrial pathway stimulation, including caspase activation and dysregulation of calcium homeostasis, resulting in atrophy and loss of DRG cells [68,69]. The data from studies in vitro or in laboratory animals showing a hypotrophy of the DRG along with neuronal atrophy seem to be contrary to the hypertrophy of DRG found in MRI in patients treated with oxaliplatin [70]. However, the differences observed in the structural abnormalities of patients’ DRG versus laboratory animals’ DRG may depend on the moment of assessment and time after oxaliplatin treatment.

### 2.2. Intracellular Signaling

Impairment in the physiological function of mitochondria may influence calcium signaling pathways and promote further pathological functional and structural changes in neuronal and glial cells. Mitochondrial and endoplasmic reticulum integrity, as intracellular stores of Ca^2+^, are crucial for Ca^2+^ homeostasis, since changes in the intracellular Ca^2+^ concentration may influence membrane excitability, neurotransmitter release and gene expression of neuronal and glial cells [71]. An increase in the intracellular Ca^2+^ concentration may result in calpain (potent protease) activation, which leads to unregulated proteolysis, directly triggering axon degeneration [72]. The oxaliplatin metabolite oxalate is a Ca^2+^ chelator and is likely involved in the pathogenesis of OIPN. The chelation of extracellular Ca^2+^ ions leads to an increase in Na+ conductance and a reduction in the threshold potential and membrane resistance [34], resulting in the early phase of cold allodynia but not late mechanical allodynia. [73]. The activation of protein kinases and caspases by chemotherapeutics may result in damage to intracellular structures. Cisplatin and oxaliplatin can also produce MAPK-related apoptosis in DRG neurons, and MAPK inhibitors may prevent DRG damage induced by platin-based agents in vitro [74]. The newly discovered role of adenosine kinase in OIPN is described further in Section 2.4, Glial Cells.

### 2.3. Ion Channels

Disturbances in the neuronal and glial functioning (membrane excitability and release of neurotransmitters), resulting clinically in the development of peripheral neuropathy, may be partially induced by the altered action of sodium channels (NaV), potassium channels (KV) and transient receptor potential (TRP) channels. The voltage-gated sodium channels are necessary to facilitate the initiation and propagation of action potentials in neurons and other excitable cells. Mutations in the genes SCNA (sodium voltage-gated channel alpha subunit) encoding proteins forming NaV channels lead to various diseases of the central and peripheral nervous systems, i.e., primary erythromelalgia, small fiber neuropathy or insensitivity to pain [75]. Palugulla et al. confirmed that the presence of polymorphisms in genes SCN4A (rs2302237), SCN9A (rs6746030) and SCN10A (rs12632942) predicts either the development or the severity of chronic OIPN in cancer patients, while patients with mutations in the SCN9A rs6754031 variant allele have a lower risk of severe chronic peripheral neuropathy development. [44]. A study performed by Sittl et al. showed an increase in the Na+ current in rodent peripheral axons and DRG neurons, with isoform NaV1.6 being involved in the development of oxaliplatin-induced cold allodynia [76]. Altered NaV channel function induced by oxaliplatin has been observed and confirmed further in numerous experimental in vivo and in vitro studies. [77,78].

Oxaliplatin injected directly into the rat hind paw leads to intense, short-duration mechanical and cold allodynia, which has been suggested to be a direct action of oxaliplatin on NaV channels on peripheral nerves but not on NaV channels in brain slices in vitro [79]. Experimental studies in vivo confirmed the involvement of NaV1.7 channels [80] as well as NaV1.9 channels [81] in OIPN. In a study by Heide et al., oxaliplatin induced the reversible slowing of sodium channel inactivation in motor axons, and these changes were strictly related to reversible cold allodynia [82]. Acute OIPN observed in clinical practice may be considered a cold-related acute channelopathy, mainly related to NaV channels, as not only paresthesia but also muscle spasms and cramps are present, which could be attributed to disturbances in NaV channel properties in both neurons and muscle cells [83].

Potassium (KV) channels are also involved in OIPN development, which has been shown in the study of Descoeur et al. and subsequently confirmed in a study by Poupon et al. [84,85]. A single administration of oxaliplatin to mice induced neuronal hyperexcitability, decreasing the expression of potassium channels, TREK1 and TRAAK (members of the two-pore domain potassium channels K2P subfamily) in DRG neurons. The oxaliplatin-induced downregulation of KV channels in cortical and DRG neurons in vitro was shown in a study by Thibault et al., which might contribute to the enhanced neuronal membrane excitability [86]. In a recent study by Viatchenko-Karpinski et al. [55], it was shown for the first time that oxaliplatin leads to a significant downregulation of the KV4.3 channel expression in trigeminal V2 neurons. These changes in KV4.3 channels resulted in an increase in membrane excitability, which could explain the cold allodynia in the orofacial region observed in cancer patients.

Transient receptor potential (TRP) channels are nonselective cation channels that detect a vast array of signals (thermal, mechanical and chemical). TRP channels play an important role in DRG neurons and, thus, may be involved in the pathogenesis of OIPN. The TRPA1 (TRP ankyrin), TRPV1 (TRP vanilloid) and TRPM8 (TRP melastatin) channels are expressed in DRG neurons, and a few preclinical studies have shown that they play a crucial role in cold and mechanical sensitivity induced by oxaliplatin and cisplatin. Exposure to oxaliplatin and cisplatin results in the altered expression and function of TRPV1, TRPA1 and TRPM8 channels in DRG neurons of laboratory animals [87,88]. TRPA1 channels can be activated by noxious cold (<18 °C) stimuli, and oxaliplatin may lead first to TRPA1 sensitization to reactive oxygen species (ROS) by hypoxia and cytosolic acidification and, after a few days, to the overexpression of TRPA1 mRNA in small DRG neurons [87,89,90,91,92,93]. These functional and structural mechanisms might highly contribute to acute and chronic cold allodynia and hyperalgesia induced by oxaliplatin, as observed in clinical practice. Oxaliplatin-induced cytosolic acidification is thought to be one of the key factors involved in the modulation of TRPA1 channels and subsequent acute cold hypersensitivity [94]. In a study by Riva et al., oxalate surprisingly reversed the pH reduction and cisplatin-induced acidification but at 100-fold higher concentrations in comparison with oxaliplatin [94]. TRPM8 channels are activated by cool temperature (<25 °C), menthol and icilin and are highly expressed in DRG neurons in laboratory animals with oxaliplatin-induced cold hyperalgesia [87,95]. The TRPV1 channels are activated by noxious heat (>43 °C), cations, lipids and capsaicin and are overexpressed after neuronal injury and inflammation. Oxaliplatin can increase the expression of TRPV1 in small DRG neurons in rats, which may contribute to the development of mechanical allodynia and thermal hyperalgesia in the chronic form of OIPN [87,96].

### 2.4. Glial Cells

Recent studies indicate that glial cells may also contribute to peripheral neuropathy induced by platinum-based agents in animal models; however, it is not clear whether this mechanism may also be involved in chemotherapy-induced neuropathy in cancer patients [97,98,99]. Oxaliplatin can activate astrocytes, and the reduction of activation by minocycline decreases the intensity of neuropathic pain behavior in rats [100].

Adenosine is a potent neuroprotective agent. Adenosine signaling at its adenosine receptors (ARs) is driven by adenosine kinase (ADK) in astrocytes. In a study by Wahlman et al., oxaliplatin in rodents caused ADK overexpression in activated astrocytes and reduced adenosine signaling at the A3AR subtype (A3AR) within the spinal cord. The dysregulation of adenosine signaling was associated with an increased proinflammatory and neuroexcitatory interleukin-1β expression [101]. These results confirm the involvement of glial cells in the pathogenesis of OIPN.

Interestingly, oxaliplatin in an in vitro study did not activate microglia but surprisingly reduced the number of microglial cells [102]. The results from experimental studies are still not consistent because the activation of microglial cells has been observed in another study [103]. In a study by Imai et al., it was shown that cisplatin and oxaliplatin may induce mitochondrial dysfunction in cultured Schwann cells, followed by numerous disturbances in molecular function and cellular structure contributing to peripheral neuropathy [64,104].

### 2.5. Inflammatory Mediators—Cytokines and Chemokines

The results from studies in human neuropathic pain and experimental animal models clearly show that the activation of glial cells and the subsequent release and elevation of pro-inflammatory cytokines (PIC): The IL-1b, IL-6 and TNF-a levels are common mechanisms of neuropathic pain induced by chemotherapeutics [99,105,106,107,108,109]. The release of cytokines induced by chemotherapy may be related to the ability of these agents to activate the Toll-like receptor (TLR) family, especially TLR4. In knockout mice lacking that receptor, the pain behavior induced by cisplatin was decreased [110]. Pro-inflammatory cytokines can lead to the sensitization of nociceptors by the modulation of ion channel properties, which has been confirmed in the study of Jin et al. [111].

Pro-inflammatory cytokines released from glial cells not only act in the peripheral nervous system but also at the spinal and supraspinal levels. In the study of Xu et al., the administration of oxaliplatin induced the activation of proinflammatory cytokines (PIC): IL-1b, IL-6, and TNF-α and their receptors in periaqueductal gray matter (PAG). Blocking the PIC receptors decreased neuropathic pain behavior induced by the administration of oxaliplatin. Additionally, PIC decreased the activity of GABA (gamma-aminobutyric acid)-ergic-mediated descending inhibition, probably by the damage of neuronal cells expressing GABA within periaqueductal gray matter (PAG) in the process of apoptosis. GABA is one of the most potent antinociceptive neurotransmitters, and a correct GABA transmission attenuated the mechanical and cold allodynia in this study. An enhanced release of PIC and subsequent decrease of GABA transmission in PAG are likely to contribute to the development of mechanical and cold hypersensitivity in oxaliplatin-treated animals [112].

Chemokines and their receptors are also involved in the pathogenesis of chemotherapy-induced peripheral neuropathy [113]. CCLs (CC chemokine ligands) are responsible for the migration and infiltration of monocytes/macrophages and other immune cells, thus contributing to neuroinflammation and pain behavior in animal models [102]. Oxaliplatin can increase the level of the CCL-2 chemokine, primarily released from astrocytes, and the level of CCL-2 is correlated with the degree of hyperalgesia observed in rats [114]. The involvement of CCL-2 and its receptor CCR2 in neuropathy induced by oxaliplatin has been confirmed in the experimental study of Illias et al. CCL2 and its receptor CCR2 were increased in the DRG after a single oxaliplatin administration and in parallel with the development of mechanical hypersensitivity [115]. CCL2 increases the sensitivity of neurons in other models of neuropathic pain, and probably the same mechanism can be observed in neuropathy induced by chemotherapeutics [116]. Other studies have confirmed the role of chemokine CXCL12 [117] and chemokine CX3CL1 signaling in OIPN [118,119]. The hyperexcitability of DRG neurons likely arises from the direct effect of CX3CL1 signaling on the function of ion channels in DRG neurons [118].

### 2.6. Central Mechanisms

In recent years, many studies have shown that chemotherapy in cancer patients can influence their cognitive and motor functioning. These data resulted in the description of a new neuropsychological syndrome associated with cancer treatment/chemotherapy-induced cognitive impairment [120,121,122,123]. Whether the abnormalities in the central nervous system induced by chemotherapeutics may contribute to peripheral neuropathy development and maintenance in humans is still unclear.

The blood–brain barrier (BBB) has been thought to prevent the access of oxaliplatin to the brain [124]; however, a direct action of oxaliplatin on BBB endothelial cells (EC) has not been ruled out. The possible mechanisms of BBB damage may include proinflammatory cytokines, ROS or other neurotransmitters, all of which are involved in the peripheral nervous system toxicity induced by chemotherapeutics [125,126]. In the study of Branca et al. [127], oxaliplatin administration in vitro induced significant changes in the junctional and cytoskeletal apparatus of endothelial cells, and these alterations of BBB may be associated with higher concentrations of oxaliplatin in the brain and probably contribute to pain chronification. The study of Sanna et al. shows that oxaliplatin administration induces changes in the levels of proteins in spinal and supraspinal levels in laboratory animals and suggests a direct correlation between structural changes in the central nervous system and chemotherapy-induced neurotoxicity [128].

## 3. Immunomodulatory Drugs (Thalidomide)

Thalidomide is a glutamic acid derivative and immunomodulatory drug that is approved by the US Food and Drug Administration for the treatment of multiple myeloma [129]. The anticancer mechanism of immunomodulatory drugs is poorly understood but may include the blocking of the production of tumor necrosis factor alpha (TNF-α), the blocking of the activation of NF-kB (nuclear factor kappaB) and the subsequent acceleration of neuronal cell death [130]. The second crucial anticancer mechanism of thalidomide is its antiangiogenic effect by blocking angiogenesis through the inhibition of basic fibroblast growth factor (b-FGF) and vascular endothelial growth factor (VEGF) [131]. Although the thalidomide effectiveness in multiple myeloma patients improves the malignancy treatment outcome, thalidomide-induced side effects may decrease the patients’ quality of life.

Thalidomide-induced peripheral neuropathy (TIPN) occurs in 25–75% of patients, with dose-dependent prevalence and severity [132]. The risk of neurotoxicity increases in a dose-dependent manner up to a cumulative dose of 20 g. In practice, thalidomide is administered at a maximum dose of 200 mg daily and for a limited treatment duration [133].

In addition to the classical sensory symptoms and signs of peripheral neuropathy (stocking and glove distribution), in severe cases of TIPN, thalidomide may induce motor impairment and gastrointestinal and cardiovascular autonomic manifestations as well [132]. In approximately 15% of patients, TIPN may lead to treatment discontinuation [134].

The data on TIPN prevalence also comes from studies on treatments with thalidomide in patients with inflammatory diseases. In the pediatric population with inflammatory bowel disease, TIPN was found in 72.5% of patients, but the prevalence depended on the time after treatment; thus, the percentage of TIPN-free patients was 70.0% at 12 months and 35.6% at 24 months of treatment. The risk of TIPN increased parallel to the mean daily dose, and TIPN was the cause of drug discontinuation in 41.8% of patients. These data confirm a dose- and time-dependent manner of TIPN development [135].

Risk factors for TIPN in multiple myeloma patients include advanced age, prior neuropathy caused by myeloma by itself or other drugs [132]. The data on the role of genetics in predicting the risk of TIPN are not consistent [136], although in the clinical study of Johnson et al., some genetic susceptibility has been proposed: the ADME gene group (drug Absorption, Distribution, Metabolism and Excretion), cytochromes, solute carrier family genes, and genes involved in neural processes and central nervous system development. It has also been suggested that the risk of TIPN is associated with single nucleotide polymorphisms (SNPs) in genes responsible for repair mechanisms and inflammation in the peripheral nervous system [137].

The pathophysiology of TIPN remains not fully understood, but it has been proposed that the antiinflammatory effect of thalidomide may partially prevent neurotoxicity. This effect has been observed in a study by Badros et al. [138], where thalidomide was neuroprotective in patients receiving it in combination with bortezomib. Since the anticancer mechanism of action of thalidomide is an antiangiogenic effect, this process is also proposed to be responsible for the secondary ischemia and hypoxia of nerve fibers, followed by irreversible damage of sensory neurons [139,140].

**Figure 2:**
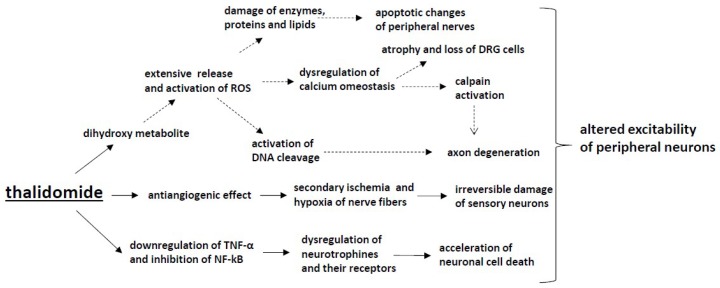
The mechanisms of CIPN induced by thalidomide: Thalidomide downregulates TNF-α and inhibits NF-κB, which leads to the dysregulation of neurotrophins and their receptors and, in consequence, accelerates neuronal cell death. Moreover, the antiangiogenic effect induced by thalidomide causes secondary ischemia and hypoxia of nerve fibres and, subsequently, irreversible damage of sensory neurons. The activation of the dihydroxy metabolite of thalidomide causes the extensive release and activation of ROS and activates DNA cleavage, though further preclinical and clinical trials are needed to confirm the presence of such a mechanism in thalidomide-induced peripheral neuropathy.

It has been proposed that the immunomodulatory effect can also contribute to TIPN (Figure 2). Thalidomide may reduce neuronal cell survival by the downregulation of TNF-α and the inhibition of NF-kB, resulting in the dysregulation of neurotrophins and their receptors and the subsequent acceleration of neuronal cell death [141]. However, in experimental studies in a neuropathic pain model, the administration of thalidomide decreased mechanical hyperalgesia after injury of the sciatic nerve in mice by reducing TNF-α levels in the sciatic nerve, prefrontal cortex and hippocampus; thus, the mechanism of TIPN may be related to other processes induced by thalidomide [142].

In a study by Wani et al., it was shown that the dihydroxy metabolite of thalidomide was capable of causing extensive redox-activated DNA cleavage, and this mechanism has been proposed to be crucial for thalidomide-induced teratogenesis. DNA cleavage was mediated through the formation of ROS, as discussed previously. ROS-dependent mechanisms have been confirmed in platinum-based chemotherapeutic neurotoxicity development, but further preclinical and clinical trials are needed to confirm this mechanism in TIPN [143].

## 4. Taxanes

Taxanes constitute a class of antineoplastic drugs acting on microtubules, interfering with the normal cycling of microtubule depolymerization and repolymerization, which causes impairment of cancer cell division and consequently leads to cell death. This class includes paclitaxel, docetaxel and cabazitaxel. They have been approved by the FDA for the treatment of various cancer types, including ovarian cancer, breast cancer, non-small cell lung cancer and prostate cancer [144].

The incidence of CIPN from taxanes may be very high and ranges from 11 to 87%, with the highest rates reported for paclitaxel [6,145]. Neuropathy caused by taxanes usually presents as a sensory dominant neuropathy, mostly affecting small diameter sensory fibers, manifesting usually as paresthesias, dysesthesias, numbness, altered proprioception and loss of dexterity predominantly in the toes and fingers (stocking-and-glove distribution); however, other localizations may appear. Motor and autonomic involvement is less frequent but may also develop [146]. The symptoms may start days after the first dose. They are dose dependent and tend to improve after stopping the treatment. In some patients, symptoms can continue up to 1–3 years after completion of the therapy and can sometimes last lifelong [147].

**Figure 3:**
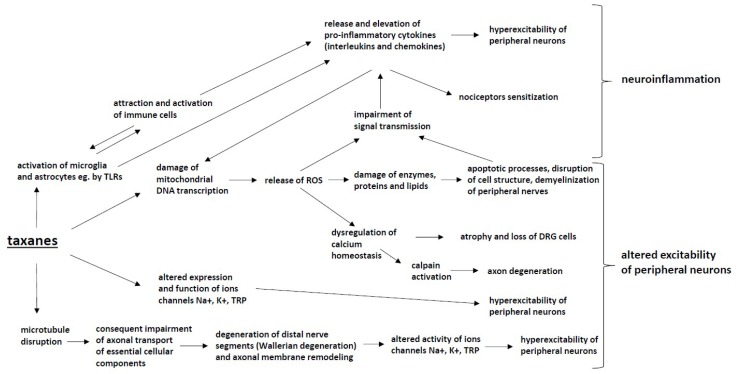
The mechanisms of CIPN induced by taxanes: Taxanes cause microtubule disruption, which impairs axonal transport and leads to Wallerian degeneration, altered activity of ion channels and hyperexcitability of peripheral neurons. Taxanes also modify the expression and function of Na+, K+ and TRP ion channels, which results in the hyperexcitability of peripheral neurons. Taxane-induced mitochondrial damage contributes to the increased production of reactive oxygen species (ROS), which leads to enzyme, protein and lipid damage as well as the dysregulation of calcium homeostasis within neurons, which induces apoptotic changes and the demyelination of peripheral nerves. These processes alter the excitability of peripheral neurons. The activation of microglia and astrocytes by taxanes also leads to the activation of attraction and activation of immune cells and to the release and elevation of pro-inflammatory cytokines (interleukins and chemokines), which results in the nociceptor sensitization and hyperexcitability of peripheral neurons. These processes lead to nociceptor sensitization and the development of neuroinflammation.

Such symptoms are most intense for paclitaxel. The docetaxel intensity of symptoms is milder [5]. Protein bound paclitaxel (Nab-paclitaxel), developed to reduce overall toxicity, does not yield a reduced incidence of CIPN [148]. The mechanisms of neurotoxicity of taxanes (Figure 3) are multifactorial and include the following pathways:

### 4.1. Microtubule Disruption

Microtubule disruption is a principal mechanism of action of taxanes and is responsible for their antineoplastic activity; however, it is also associated with the development of CIPN [149]. The aggregation and bundling of microtubules lead to changes in cell shape and cell stability but are also responsible for the impairment of axonal transport of synaptic vesicles loaded with essential cellular components, including lipids, proteins and ion channels [150,151,152].

### 4.2. Mitochondrial Dysfunction

Damage to mitochondria, both in neuronal and nonneuronal cells, leads to oxidative stress and the production of reactive oxygen species (ROS), such as hydroxyl radicals, peroxide, superoxide and single oxygen. Impaired axonal transport of essential cellular components [151,153] and mRNA [154] to distal neuronal parts due to microtubule disruption may have a significant impact on this process. Increased levels of ROS have been detected in sensory neurons and the spinal cord [23,62,155,156,157]. Increased ROS levels cause the activation of apoptotic processes, the disruption of cell structure and demyelinization. These events lead to the impairment of signal transmission and the activation of immune processes, including increased production of pro-inflammatory cytokines. The process is self-amplifying as the above mechanisms can cause further mitochondrial damage [23,158,159,160]. Swelling, vacuolation and loss of structure of mitochondria have been proven in a number of studies with paclitaxel [161,162].

### 4.3. Axon Degeneration

The direct damage of peripheral nerves, the loss of neuronal fibers and demyelinization caused by paclitaxel have been reported in various studies [149,163,164,165,166]. Microtubule disruption and the consequently impaired axonal transport of essential cellular components cause the degeneration of distal nerve segments (Wallerian degeneration) and axonal membrane remodeling [167]. Boyette et al. showed a decreased number of intraepidermal fibers in a rodent model of paclitaxel-induced CIPN [168]. Ferrari et al. described impaired corneal innervation in rats. Cytokine and chemokine signaling may also play a role in axon degeneration. Zhang et al. showed that a decrease in the level of chemokine MCP1/CCL-2 decreases nerve degeneration and CIPN behaviors in a rodent model [169].

### 4.4. Altered Calcium Homeostasis

The dysregulation of Ca^2+^ hemostasis has been shown to play a role in the pathogenesis of CIPN [170]. The dysregulation of intracellular Ca^2+^ was observed in a paclitaxel neuropathy model in both neuronal and nonneuronal cells [171,172,173]. Mitochondria and endoplasmic reticulum (ER) are intracellular magazines of Ca^2+^. Paclitaxel can cause the release of Ca^2+^ from mitochondria, and the process is probably mediated by the activation of the mitochondrial permeability transition pore (mPTP), leading to rapid mitochondria depolarization [172,173]. Paclitaxel can probably also stimulate the release of Ca^2+^ from the ER; this process may be mediated by the 1,4,5-trisphosphate receptor (IP3R) [174,175], leading to the increased expression of CaV3.2 channels in rats, with a suppression of these channels reversing hyperalgesia [176].

### 4.5. Changes in Peripheral Nerve Excitability

The altered expression and function of ion channels (NaV, KV and TPR) is another mechanism contributing to the development of CIPN. The decreased expression of K^+^ channels causing the spontaneous activity of nociceptors was observed in the DRG in a paclitaxel-induced CIPN model [177]. The activation of cation channels TRPV1 and TRPA1, important components of pain signaling, was detected in DRG neurons [178,179]. Antagonists of TRPA1 have been shown to relieve inflammation, cold allodynia and hyperalgesia induced by paclitaxel [180]. Paclitaxel treatment increases the number of NaV1.7 channels, which may be responsible for the development of CIPN [181,182]. Gheraldin et al. [80] showed that blocking this channel attenuates hyperalgesia in rats.

### 4.6. Immune Processes and Neuroinflammation

Paclitaxel causes an increase in the production of pro-inflammatory cytokines (TNF alfa and IL-1 beta) and a decrease in anti-inflammatory cytokines (IL-4 and IL-10) [23,155]. This process leads to the attraction and activation of immune cells and the development of neuroinflammation [183]. Krukowski et al. [184] showed that IL-10 can attenuate paclitaxel-induced CIPN. Paclitaxel can also lead to microglial and astrocyte activation [185,186] and an increase in macrophage number in DRG and peripheral nerves [187]. The inhibition of macrophages and microglia prevents the development of mechanical hyperalgesia and epidermal nerve fiber loss [187,188]. The release of cytokines stimulates the TLR4 receptor in DRG cells, and blocking this receptor decreases pain behaviors in mice [189].

## 5. Epothilones (Ixabepilone)

Epothilones, represented mainly by ixabepilone, an analog of epothilone B and sagopilone are relatively new antineoplastic drugs acting with similar mechanisms to taxanes as tubulin destabilizers and thus preventing the division of cancer cells. These drugs bind preferentially to the beta III tubulin isotype. Ixabepilone is approved by the FDA in the US but not in Europe for the treatment of breast cancer not responding to other available chemotherapies [190]. Sagopilone is used to treat various cancers, including non-small cell lung cancer, ovarian cancer and prostate cancer, but it does not have FDA approval at present [191]. The prevalence of CIPN for ixabepilone has been estimated to be approximately 67%, with an incidence of severe CIPN ranging from 1% in previously untreated patients to 24% of patients already treated with other chemotherapeutics. As ixabepilone is registered for patients not responding to other available chemotherapies, the incidence is usually within the higher ranges. As the mechanism of action of epothilones is similar to that of taxanes, the clinical manifestation of CIPN is similar for these two classes of drugs [192].

The clinical manifestation of neuropathy caused by epothilones presents as mild or moderate sensory dominant neuropathy, mostly affecting small diameter sensory fibers, usually manifesting as paresthesias, numbness and pain in the feet and hands (stocking-and-glove distribution); however, other localizations may also appear. Motor involvement is less frequent but possible. Usually, it is preceded by sensory neuropathy and can manifest as mild muscle weakness, i.e., difficulty in climbing stairs. Autonomic manifestation is very rare and is observed in less than 1% of patients. The symptoms are dose-dependent and tend to improve after stopping the treatment [192].

**Figure 4:**
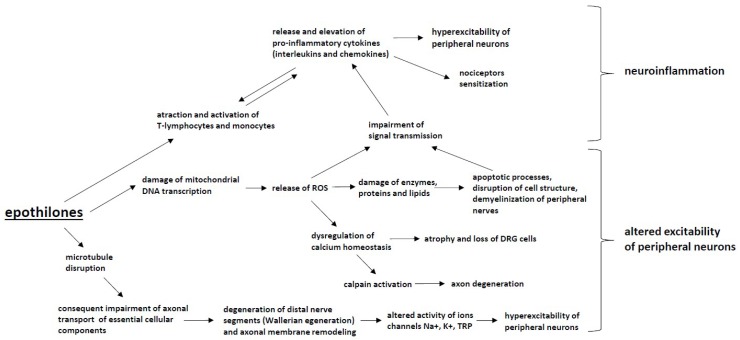
The mechanisms of CIPN induced by epothilones: Epothilones cause microtubule disruption, which impairs axonal transport and leads to Wallerian degeneration, the altered activity of ion channels and the hyperexcitability of peripheral neurons. Furthermore, the damage to mitochondria by epothilones leads to an increased production of reactive oxygen species (ROS), resulting in enzyme, protein and lipid damage within neurons as well as apoptotic changes to peripheral nerve. These processes lead to altered excitability of peripheral neurons. ROS release and the attraction and activation of T-lymphocytes and monocytes also induces the release and elevation of pro-inflammatory cytokines (interleukins and chemokines), the activation of immune cells and the development of neuroinflammation.

Since epothilones constitute a relatively new class of antineoplastic drugs, the number of studies regarding epothilone-induced CIPN is very limited. The mechanisms of the neurotoxicity of epothilones remain unclear (Figure 4). Some of the pathomechanisms are shared with those of taxanes due to a similar primary mechanism of action targeting microtubule disruption [192]. 

Mitochondrial dysfunction associated with oxidative stress also contributes to the development of CIPN for this group of chemotherapeutic agents [193]. However, the severity of CIPN associated with epothilones is smaller than that of taxanes [192], and it is postulated that some of the off-target mechanisms associated with the development of taxane-induced neuropathy may not be shared for epothilones. However, data regarding this issue are limited, and more studies are needed to understand the pathomechanism of epothilone-induced CIPN.

## 6. Vinca Alkaloids

**Figure 5:**
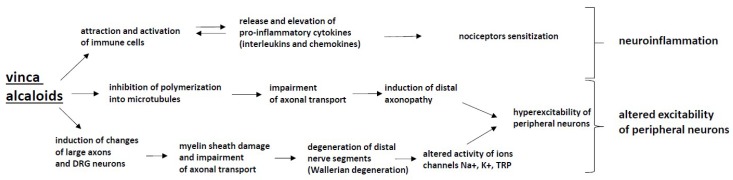
The mechanisms of CIPN induced by vinca alkaloids: Vinca alkaloids cause changes to large axons and DRG neurons, which leads to Wallerian degeneration, the altered activity of ion channels and the hyperexcitability of peripheral neurons. Moreover, the inhibition of polymerization into microtubules inhibits axonal transport, which leads to distal axonopathy. These processes alter the excitability of peripheral neurons, whereas the attraction and activation of immune cells by vinca alkaloids causes the release and elevation of pro-inflammatory cytokines (interleukins and chemokines), which results in neuroinflammation.

Vinca alkaloids are drugs developed from the Madagascar periwinkle plant and include vincristine, vinblastine, vinorelbine and vindesine. The group is commonly used to treat various tumors, such as Hodgkin and non–Hodgkin lymphoma, testicular cancer and non–small cell lung cancer. The vinca alkaloids inhibit the assembly of microtubules and promote their disassembly, thus disrupting axonal transport (Figure 5). Although vinca alkaloids do not readily cross the blood–brain barrier, they can act on the cell body of peripheral nerves. All vinca alkaloids induce sensorimotor neuropathy that is dose–dependent. Symptoms usually appear within the first 3 months of treatment. Pain located in hands and feet may be early symptoms. Other symptoms include muscle weakness, including wrist extensors, and dorsiflexor weakness and cramping [194].

The most neurotoxic drug from the group of vinca alkaloids is vincristine. The neurotoxic effects of vincristine are observed at cumulative doses of 4 mg/m^2^. Vincristine induces axonal neuropathy by disrupting the microtubular axonal transport system, as a reduced compound muscle potential and sensory nerve action potential amplitude are observed. Vincristine induces distal actional degeneration, and vincristine–induced peripheral neuropathy is associated with pain [195].

Vincristine binds with tubulin and blocks its polymerization into microtubules. By binding intracellular tubulin and altering cellular microtubular structures, vincristine inhibits both fast and slow actional transport, which may induce distal axonopathy. Other changes induced by vincristine include ultrastructural changes in the cytoskeleton of large myelinated axons and an accumulation of neurofilaments in dorsal sensory ganglion neurons [5].

Vincristine may produce the most severe neuropathy, with symptoms of distal numbness and tingling commonly beginning approximately 4–5 weeks after treatment. The neuropathy tends to involve both motor and sensory fibers; small fiber modalities are notably affected. Autonomic fibers are also affected. Distal weakness may occur rapidly and limit further treatment [196].

Vinca alkaloids bind to tubulin, thereby inhibiting the formation of microtubules and blocking cell division. Impaired axonal transport is also implicated, likely resulting from cytoskeletal disorganization. Neurotoxicity can occur at doses of 1.4 mg/m^2^ per week [197] with sensory symptoms and painful paresthesias arising first, and distal weakness typically occurs after doses above 6–8 mg/m^2^. Autonomic symptoms occur in up to one-third of patients. The symptoms and incidence of neuropathy increase with increased cumulative doses and with more frequent dosing [198].

Among the genetic factors that may affect neuropathy severity, Charcot–Marie–Tooth disease type 1A (CMT1A) and vincristine should be mentioned—a single dose can transform an asymptomatic carrier to significant weakness [199]. The FDA issued a black box warning on vincristine use in Charcot–Marie–Tooth disease (CMT) patients. CMT patients with the ERG2 gene mutation are more sensitive to vincristine [200]. Polymorphism in the CEP72 gene is associated with an increased risk and severity of vincristine-induced neuropathy [201].

Among possible preventive measures, tropisetron, a 5–HT3 receptor antagonist that significantly suppresses vincristine–related neuropathy in a rat model, should be mentioned [202]. Genes that reduce Wallerian degeneration are also known, notably the WldS (slow Wallerian degeneration gene). Most recently, the deletion of SARM1 (sterile alpha and TIR motif–containing protein 1) was found to protect mice against both axotomy and vincristine exposure compared to the corresponding effects in control animals [203].

## 7. Protease Inhibitors: Bortezomib

Bortezomib and carfilzomib are reversible proteasome inhibitors used for the treatment of multiple myeloma and certain types of lymphoma. Sensory neuropathy is very painful [204]. Sometimes weakness with demyelinating neuropathy may also be present [205]. The frequency is approximately 34% of treated patients [206]. Patients receiving bortezomib develop chronic, distal and symmetrical sensory peripheral neuropathy often accompanied by a neuropathic pain syndrome that may last for weeks, months or even years after drug termination [207].

The neurotoxicity of bortezomib is dose-dependent, and a dose adjustment may be necessary with an increase in its toxicity. A length-dependent, mixed small and large fiber axonal sensory neuropathy is present and may disappear after several weeks after treatment cessation. The subcutaneous formulation of bortezomib evokes a lower incidence of neuropathy without reduced therapeutic efficacy [208,209]. The oral proteasome inhibitor ixazomib may have less toxicity.

**Figure 6:**
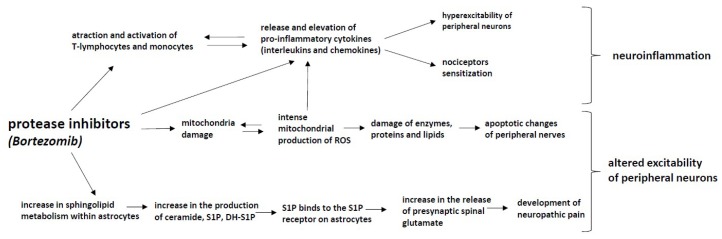
The mechanisms of CIPN induced by protease inhibitors: Protease inhibitors increase the metabolism of sphingolipids in astrocytes, which leads to the formation of ceramide, sphingosine-1 phosphate (S1P) and dihydrosphingosine-1-phosphate (DH-S1P), which by binding to astrocyte receptors, increase the release of presynaptic glutamate at the level of the dorsal horn, which leads to the development of neuropathic pain. Moreover, bortezomib-induced mitochondrial damage increases the production of reactive oxygen species (ROS), which results in enzyme, protein and lipid damage within the neurons as well as induces apoptotic changes in peripheral nerves. These processes alter the excitability of peripheral neurons, whereas the attraction and activation of T-lymphocytes and monocytes, as well as the increases in the production of reactive oxygen species (ROS) induce the release and elevation of pro-inflammatory cytokines (interleukins and chemokines). These processes lead to nociceptor sensitization, the hyperexcitability of peripheral neurons and the development of neuroinflammation.

Despite having opposing effects on cancer cells, compelling evidence is emerging that ceramide and sphingosine-1 phosphate (S1P) share potent inflammatory and nociceptive actions [210]. It is noteworthy that altered sphingolipid metabolism caused by mutations in serine palmitoyltransferase (SPT) contributes to neuropathic pain in humans [211]. Within astrocytes, bortezomib causes an increase in sphingolipid metabolism, leading to an increase in the production of ceramide as well as sphingosine-1 phosphate (S1P) and dihydrosphingosine-1-phosphate (DH-S1P). Within the periphery, bortezomib increases the production of tumor necrosis factor α (TNF-α) and interleukin-1β, which in turn act to augment sphingolipid metabolism within astrocytes. Released S1P binds to the S1P receptor (S1PR1) on astrocytes, which ultimately leads to an increase in the release of presynaptic glutamate at the level of the dorsal horn of the spinal cord and to the development of neuropathic pain (Figure 6) [212,213].

Whether sphingolipid dysregulation drives bortezomib-induced neuropathic pain is not known. S1PR1-dependent neuroinflammatory signaling pathways contribute to the development of bortezomib-induced neuropathic pain, identifying S1PR1 as a molecular target for therapeutic intervention.

Some SNPs are involved in the development of late vs. early onset bortezomib neuropathy, demonstrating the role of underlying genetic factors [214]. A locus in the PKNOX1 gene and one near the CBS gene are associated with bortezomib neuropathy [215]. The inhibition of nuclear factor kappa B (NFκB), a transcription factor involved in cell survival and proliferation, is an important mechanism of action regarding the inhibition of tumor growth and neuronal survival. In a transgenic mouse model that selectively blocks the NFκB pathway in neurons, animals with impaired NFκB activation developed significantly less severe neuropathy than wild–type animals [216]. Low levels of vitamin D are associated with a higher severity of bortezomib neuropathy in myeloma patients. Therefore, it is suggested to monitor vitamin D levels in these patients [217]. Mitochondrial damage was also shown to result from bortezomib treatment, which can lead to intense mitochondrial production of reactive oxygen species (ROS), which may, in turn, impair mitochondrial function [218].

## 8. Conclusions

Chemotherapy-induced painful neuropathy (CIPN) is a major dose-limiting side effect of several first-line chemotherapeutic agents. Given the prevalence of common cancers treated with chemotherapeutics, CIPN annually affects several million patients worldwide. Due to its high prevalence among cancer patients and negative impact on their quality of life, CIPN constitutes a major problem for both cancer patients and survivors, as well as for their health care providers.

The key questions are how to manage patients treated with potentially neurotoxic chemotherapeutics and whether it is possible to prevent and/or alleviate chemotherapy-induced neuropathy symptoms without limiting the potentially life-saving chemotherapy. Patients should be instructed to report any signs of neuropathic pain, cases of altered sensory perception and any other CIPN symptoms as soon as possible. Moreover, especially in high-risk patients, if known neurotoxic chemotherapeutics are used, a neurological examination with electrophysiological evaluation should be implemented early in the course of treatment and repeated as required by the particular clinical context. The use of neurotoxic chemotherapeutics should be closely monitored, and in the event of neurotoxicity symptoms, if clinically permitted (i.e., in the case of cancer regression), drug doses should be reduced or combined with other less neurotoxic anticancer agents.

The idea to prevent CIPN by pretreatment with appropriate agents to reduce the incidence or severity of CIPN would be the optimal option. A number of drugs have been tested specifically for this purpose. Certain compounds showed initial promise, but none of them are currently applied or recommended. Clinical practice guidelines promulgated by the American Society of Clinical Oncology (ASCO) do not recommend any agent for the prevention of CIPN. In the ASCO guidelines, for the treatment of established CIPN, a moderate recommendation was made for duloxetine. There was also a weak recommendation for a topical gel containing baclofen, amitriptyline and ketamine [7,219]. Given the limited possibilities of CIPN treatment, a better understanding of its mechanisms is needed to facilitate the development of new and effective treatment strategies. A better understanding of CIPN risk factors (including genetic factors) may help identify the most susceptible patients and may guide new methods of treatment in the future. The cooperation between preclinical and clinical researchers is necessary to translate an improved understanding of the underlying CIPN mechanisms into effective preventive and treatment strategies. Given the high prevalence of the condition among cancer patients, recent major improvements in the management of several cancer types and the increasing numbers of cancer survivors, discovering new, effective strategies to prevent and/or treat CIPN and their long-term consequences has become a matter of urgency.

It should also be emphasized that advances in genome-wide association studies (GWAS) allow for the identification of critical genes important in the pathophysiology underlying the toxicity of chemotherapeutics. Novel genes found from GWAS on CIPN have alluded to the importance of biological pathways in peripheral nerve damage [220]. A GWAS on oxaliplatin-induced neuropathy implicated genes relating to nerve development and neuron extension (FOXC1 and ITGA1) and pain signaling neurotransmitters (TAC1). The first GWAS on paclitaxel-induced peripheral neuropathy identified three novel genes important in neurite growth during development (EPHA5 and FZD3). Importantly, EPHA5 and related EPHA receptors were identified as critical genes for paclitaxel-induced CIPN. A recent GWAS of docetaxel-induced peripheral neuropathy identified a gene implicated in neurodegeneration (VAC14), and a gene related to cellular structure (CEP72) was identified in a GWAS for vincristine-induced neurotoxicity. This is important because, in the future, a translation of the results of GWAS will allow the prediction of CIPN occurrence after chemotherapeutics administration.

The recent years of studies give more evidence by which mechanisms of chemotherapeutic agents induced peripheral neuropathy, and the animal studies give hope for the possibility of polytherapy with immunomodulators among which one can find substances with a wide range of activities such as minocycline, medicinal plants and phytochemicals [221,222,223]. Unfortunately, in humans, existing evidences cannot yet support their application. Future well-designed clinical trials are needed to evaluate the usefulness, efficiency and safety of polytherapy in patients with CIPN.
